# Supplementary material for: Persistent homology elucidates hierarchical structures responsible for mechanical properties in covalent amorphous solids
Source: Nat Commun. 2025 Sep 25;16:8226. doi: 10.1038/s41467-025-63424-z (PMC12462471; doi:10.1038/s41467-025-63424-z)
Supplement: Supplementary file 1 — Supplementary Information [file 41467_2025_63424_MOESM1_ESM.pdf]

Supplementary Information for

Persistent homology elucidates hierarchical structures responsible for mechanical properties in covalent amorphous solids

Emi Minamitani*[1,2], Takenobu Nakamura[3], Ippei Obayashi[4], Hideyuki Mizuno[5]

1. SANKEN, The University of Osaka, 8-1 Mihogaoka, Ibaraki, Osaka 567-0047, Japan
2. JST, PRESTO, 4-1-8 Honcho, Kawaguchi, Saitama 332-0012, Japan
3. Department of Materials and Chemistry Materials DX Research Center, National Institute of Advanced Industrial Science and Technology (AIST), 1-1-1 Umezono, Tsukuba, Ibaraki 305-8568, Japan
4. Center for Artificial Intelligence and Mathematical Data Science, Okayama University, Okayama 700-8530, Japan
5. Graduate School of Arts and Sciences, The University of Tokyo, Tokyo 153-8902, Japan

Spatial distribution of Born term and nonaffine displacement

Supplementary Figure 1 is the visualization of the difference in the spatial distribution of the Born term, B_i, and nonaffine displacement, D_i. The atom color in the a-Si model structure corresponds to B_i and $\log(D_i)$. Atoms with low B_i do not always have large D_i (Figure 2b of the main text).

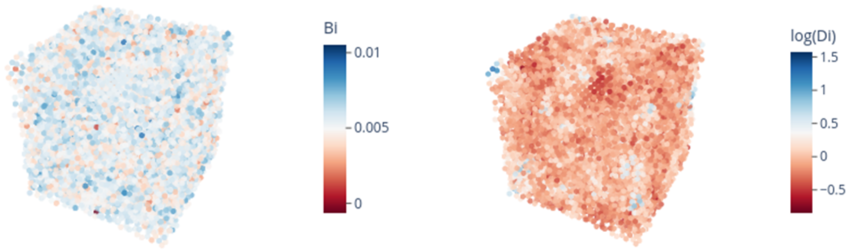

Supplementary Figure 1: a-Si structure models colored according to Born term B_i (left) and nonaffine displacement D_i (right).

Dependency on the number of atoms considered in the analysis

In the main text, we focused on 10 atoms with the largest Born terms (Bmax10), 10 atoms with the smallest Born terms (Bmin10), and 10 atoms with the largest nonaffine displacements (NAmax10). Here, we extended our analysis to include the top 20 and 50 atoms. The results of birth–death pair distribution of the ring structures that included at least one atom from the respective group are shown in Supplementary Figure 2 **a–i**. As the number of target atoms increased, the number of birth–death pairs to be considered also increased, making it harder to distinguish clear trends in the scatter plots. To compare the trend in the birth–death pair distribution quantitatively, we also analyzed the accumulated persistence function (APF) for the top 20 and 50 atoms (Supplementary Figure 2 **j–l**). In all cases, APFs exhibited a step-like shape, with the initial rise near m = 2.3 and saturation around m = 3.0, and the saturated APF values consistently followed the order Bmax > NAmax > Bmin. We also checked the variation in the APF for 10 different samples. Despite slight sample-to-sample variation, the APF values still consistently follow the order. The results using the averaged value for 10 samples are shown in Supplementary Figure 3.

The histograms of the vertex count in the ring structures also exhibited similar trends, even when the top 20 or 50 atoms were considered (Supplementary Figure 4). These results indicate that the results obtained from focusing on the top 10 atoms provides a reasonable representation of the overall

behavior.

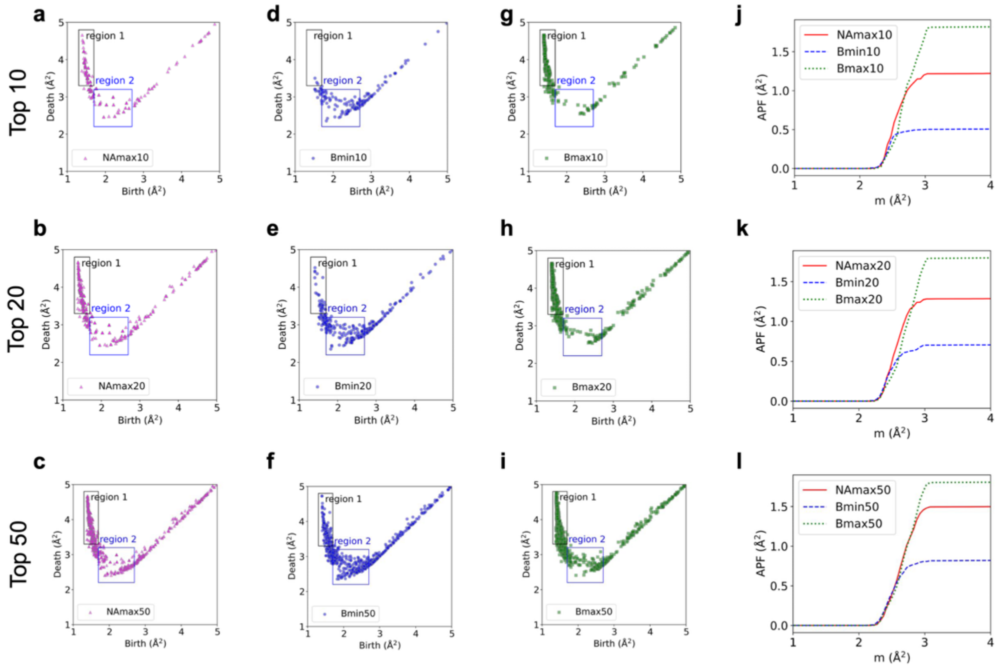

Supplementary Figure 2: Dependency on the number of atoms considered in the analysis. **a**–**i** Birth–death pair distributions for the ring structures that include at least one atom from three groups: the top 10, 20, or 50 atoms with the largest nonaffine displacements (NAmax10, NAmax20, or NAmax50 in panels **a**, **e**, and **i**, respectively); the top 10, 20, or 50 atoms with the largest Born terms (Bmax10, Bmax20, or Bmax50 in panels **b**, **f**, and **j**, respectively); and the top 10, 20, or 50 atoms with the smallest Born terms (Bmin10, Bmin20, or Bmin50 in panels **c**, **g**, and **k**, respectively). **j**–**l** Accumulated persistence functions calculated from the birth–death distributions shown in panels **a**–**i**.

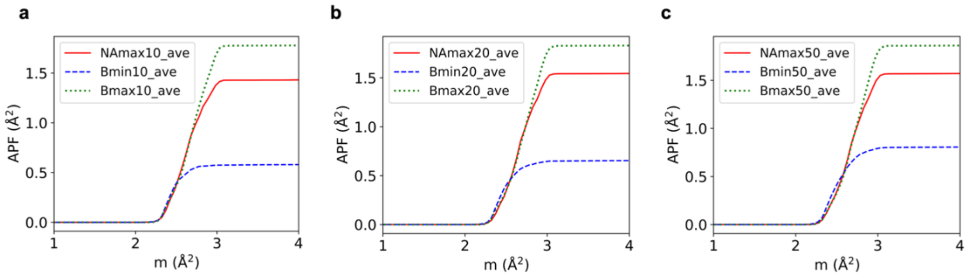

Supplementary Figure 3: Average of accumulated persistence function obtained from 10 a-Si samples. Analyses focusing on the top **a** 10, **b** 20, and **c** 50 atoms. The names NAmax, Bmin, and Bmax are the same as those in Supplementary Figure 2.

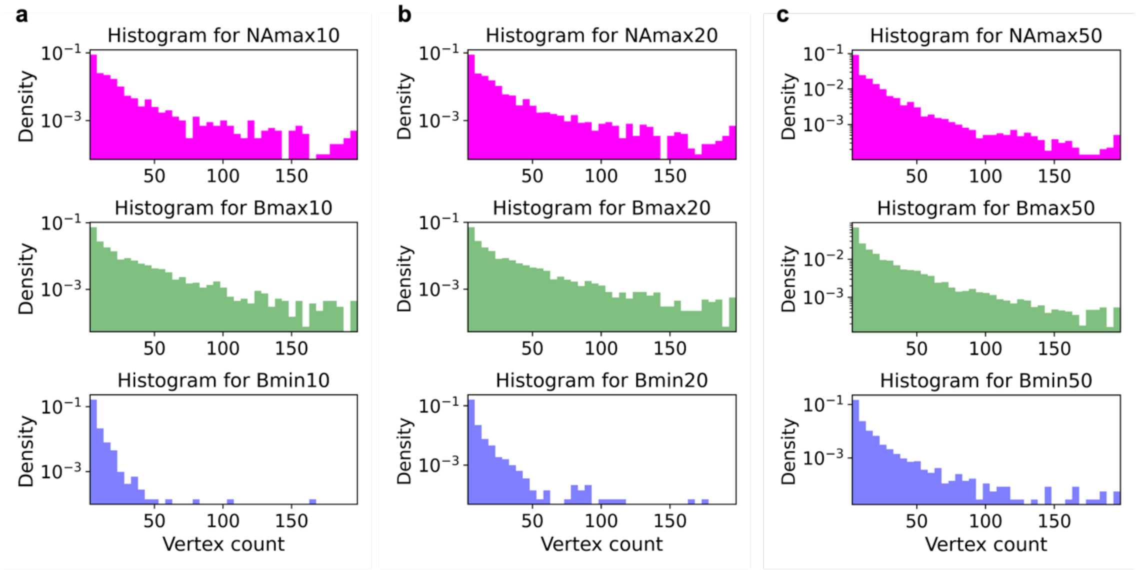

Supplementary Figure 4: Histograms of the number of vertices in ring structures corresponding to cycles analyzed separately for each group of atoms: those with large nonaffine displacement, large Born term, and small Born term. Each ring structure contains at least one atom from the respective group. Analyses focusing on the top **a** 10, **b** 20, and **c** 50 atoms. The names NAmax, Bmin, and Bmax are the same as those in Supplementary Figure 2.

Disorder in bond angles

The bond length disorder observed in region 2 in Fig. 5c is expected to extend to bond angle disorder. To investigate this, we analyzed the angular distributions within ring structures obtained from the inverse analysis of persistent homology for birth–death pairs in regions 1 and 2 of NAmax10. Our comparison was limited to five-, six-, and seven-membered rings. This restriction was necessary because the structures extracted using persistent homology are not strictly defined by atomic bonds, in contrast to conventional ring statistics based on atomic connectivity. In rings where the number of vertices deviates significantly from six, the internal angles may not correspond to physically meaningful bond angles.

Consequently, we identified 46 five-membered, 31 six-membered, and 21 seven-membered rings in region 1. In region 2, we found 27 five-membered, 31 six-membered, and 7 seven-membered rings. For each ring, we determined the presence of bonds between vertex pairs using a 2.8-Å cutoff and then constructed histograms of the resulting bond angles.

As shown in Supplementary Figure 5, the bond angle distribution for rings in region 1 exhibits a sharp peak centered at approximately 109°, which is consistent with the tetrahedral bond angle in the diamond structure of Si. In contrast, the distribution in region 2 is broader. This suggests that bond angles are also disordered in region 2, indicating the presence of short-range disorder that disrupts medium-range order.

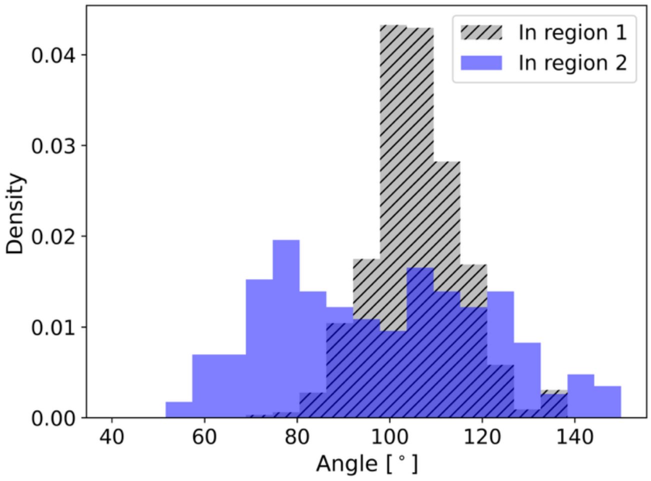

Supplementary Figure 5. Bond angle distributions in five, six, and seven-membered ring structures associated with birth-death pairs located in region 1 (hatched) and region 2 (filled).

Analysis using structural indicators proposed in previous studies

We analyzed two typical local structural indicators in the a-Si sample: the Voronoi volume and interatomic angle (Supplementary Figure 6). The Voronoi volume of each atom was not correlated with nonaffine deformation and only weakly correlated with the Born term. In the scatter plot of the average and standard deviation of interatomic angles, Bmax10 is concentrated in specific regions. Therefore, the characteristics of the atoms with large Born terms are captured by using this indicator. In contrast, NAmax10 and Bmin10 are distributed across various locations and have no clear features.

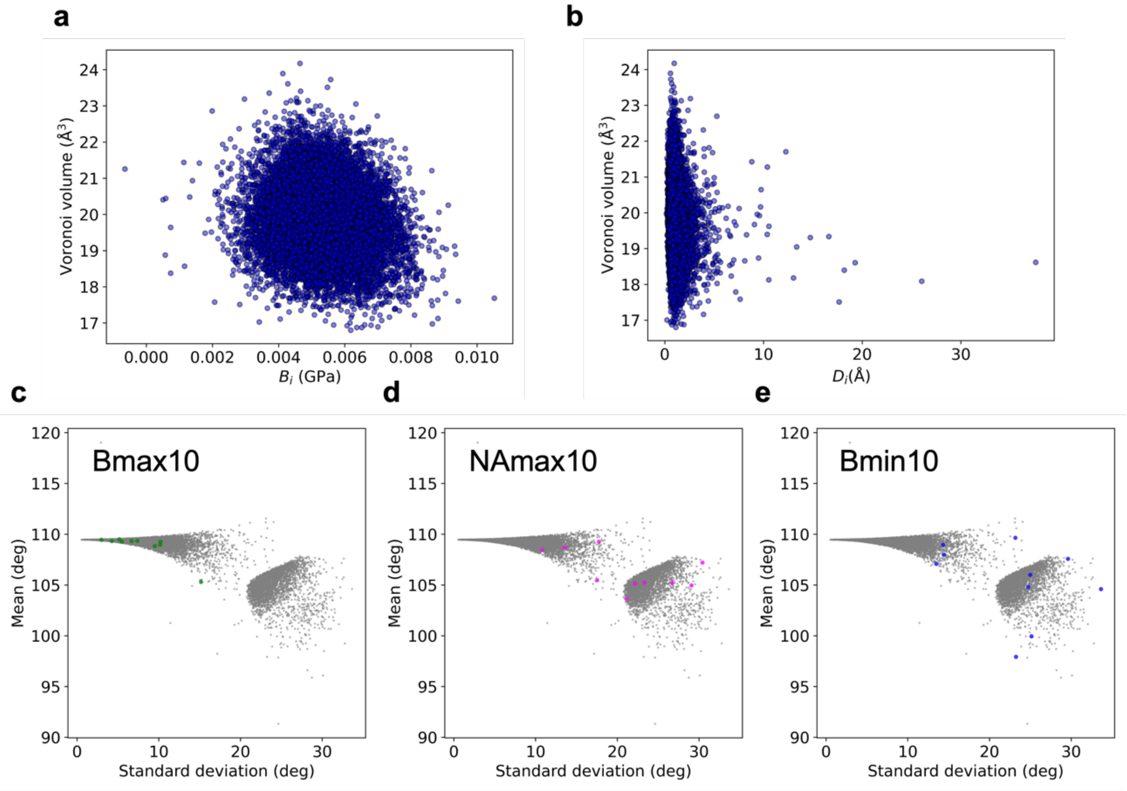

Supplementary Figure 6: Results of analysis using local structural indicators. **a** Relationship between the Voronoi volume and B_i. **b** Relationship between the Voronoi volume and D_i. **c–e** Scatter plots for mean and standard deviation of interatomic angles. The data for Bmax10, NAmax10, and Bmin10 are highlighted in green, magenta, and blue, respectively.

Participation ratio and inverse participation ratio for full modes

In addition to the results for the 1000 lowest-frequency modes, we evaluated the participation ratio (PR) and inverse participation ratio (IPR) for all modes (Supplementary Figure 7). IPR is defined as

$$IPR(p) = N_{atom} \frac{\sum_{i=1}^{N_{atom}} \left(\left(\psi_{p_{ix}}\right)^2 + \left(\psi_{p_{iy}}\right)^2 + \left(\psi_{p_{iz}}\right)^2 \right)^2}{\left(\sum_{i=1}^{N_{atom}} \left(\psi_{p_{ix}}\right)^2 + \left(\psi_{p_{iy}}\right)^2 + \left(\psi_{p_{iz}}\right)^2 \right)^2}.$$

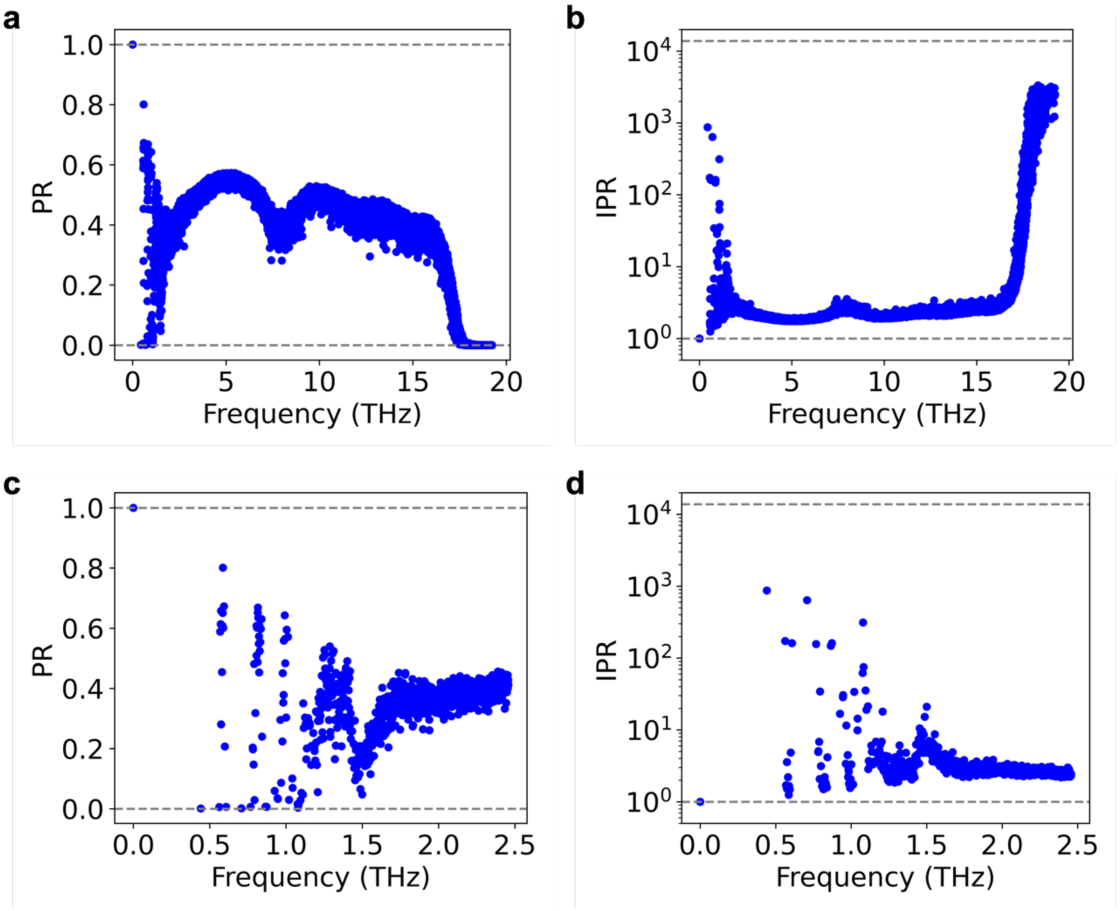

Supplementary Figure 7: Calculation results of the PR and IPR. **a, b** PR and IPR for all vibrational modes as a function of frequency. **c**, **d** Magnified views of **a** and **b**, focusing on the 1000 lowest-frequency modes. Gray dashed lines represent the upper limit (PR = 1.0, IPR = N_{atom}= 13,824) and lower limit (PR = 0, IPR = 1) of the PR and IPR.

Mode-demixing procedure

We employed the mode-demixing procedure described in Refs.[1,2] to address the hybridization between the localized and extended modes. We begin by focusing on a target localized mode, p_1, which has the smallest participation ratio $PR1$ (below 0.25) and its corresponding frequency ω_1. We then select another mode, p_2, with a frequency ω_2 that falls within a ±30% frequency range around ω_1, i.e., $\omega_2 \in [0.7\ \omega_1, 1.3\ \omega_1]$. For this pair of modes, we rotate the basis of eigenvectors by an angle θ such that the participation ratio $PR'1$ of the rotated target mode is minimized. The transformation is given by

$$\psi'_{p_1 i_\alpha} = \psi_{p_1 i_\alpha} \cos\theta + \psi_{p_2 i_\alpha} \sin\theta,$$
$$\psi'_{p_2 i_\alpha} = -\psi_{p_1 i_\alpha} \sin\theta + \psi_{p_2 i_\alpha} \cos\theta,$$

where $\psi_{p_1 i_\alpha}$ and $\psi_{p_2 i_\alpha}$ are the original eigenvectors, while $\psi'_{p_1 i_\alpha}$ and $\psi'_{p_2 i_\alpha}$ are the rotated eigenvectors. i denotes the particle index, and α represents the x, y, and z components. Notably, this transformation preserves the orthonormality of the eigenvectors.

The frequencies ω_1 and ω_2 are also modified as follows:

$$(\omega'_1)^2 = \omega_1^2 \cos^2\theta + \omega_2^2 \sin^2\theta,$$
$$(\omega'_2)^2 = \omega_1^2 \sin^2\theta + \omega_2^2 \cos^2\theta.$$

These transformed frequencies are expected to be the eigenvalues of the Hessian matrix corresponding to the rotated eigenvectors. This procedure is applied to all modes p_2 within the $\pm 30\%$ frequency range around ω_1.

After completing this process for the target localized mode p_1, we proceed to the localized mode with the second-lowest PR and apply the same procedure. This process is iteratively applied to all the localized modes with PRs below 0.25. As illustrated in Figure 8 in the main text, these demixing steps lead to a distinct separation between modes with PRs higher and lower than 0.25.

To investigate the size dependence of low-energy localized modes and its mitigation by the mode-demixing procedure, we analyzed vibrational modes in larger a-Si systems with N=21,952 and 32,768, alongside the N=13,824 system used in the main text. In the larger systems, we focused on the 2000 vibrational modes with the lowest frequencies. Supplementary Figure 8 shows the participation ratio versus the mode frequency for these larger systems, compared with the original N=13,824 system.

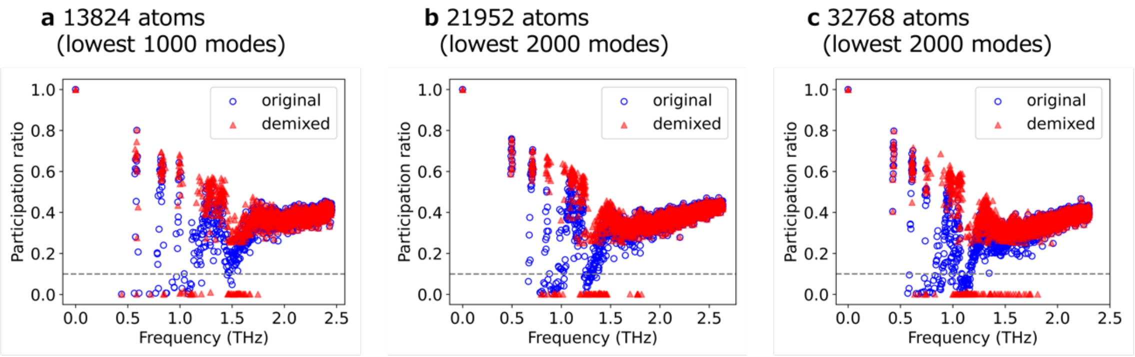

Supplementary Figure 8: Participation ratio versus mode frequency, computed from both the original and demixed eigenvectors, for three system sizes.

To explicitly assess size dependence, we plotted $N \times PR_p$ against the mode frequency ω_p, as shown in Supplementary Figure 9, where PR_p and ω_p represent the participation ratio and frequency of the p-th mode, respectively. $N \times PR_p$ represents the number of atoms contributing to each mode and is expected to scale with system size for extended modes but remain constant for localized ones, as established in prior studies[3,4].

In Supplementary Figure 9, the original eigenvectors exhibit size dependence across the entire frequency range, except for a few low-frequency localized modes. In contrast, for the eigenvectors after demixing, the extended modes exhibit an increase in $N \times PR_p$ proportional to N, while the localized modes lack systematic N dependence.

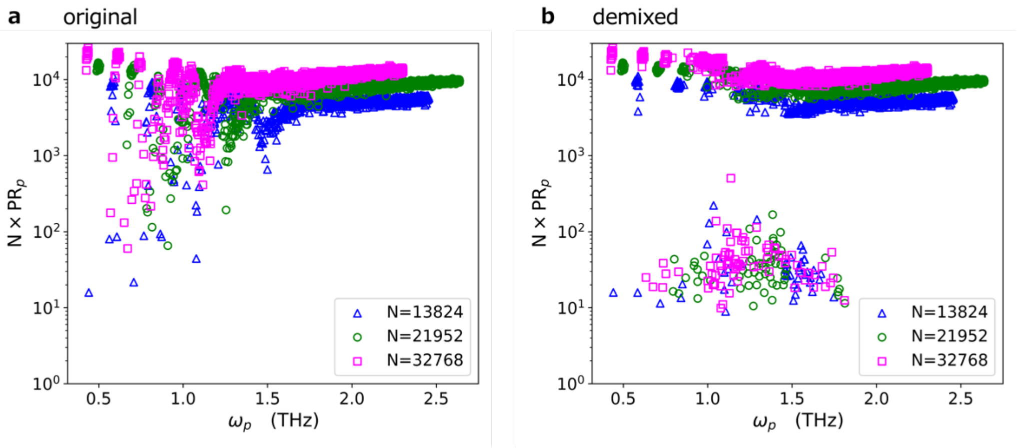

Supplementary Figure 9: $N \times PR_p$ plotted against mode frequency ω_p for three system sizes: Results obtained from **a** original and **b** demixed eigenvectors.

Correlation between localized modes and mechanical properties

We investigated the correlation between the localized modes obtained from the aforementioned mode-demixing procedure and two mechanical properties: the nonaffine displacement D_i and Born term B_i. In the main text, we discussed their correlation with AMP_i. Here, we compare AMP_i values calculated from the eigenvectors before and after demixing, as shown in Supplementary Figure 10**a**. The results reveal that D_i-AMP_i and B_i-AMP_i correlations are largely preserved between the original and demixed eigenvectors (Supplementary Figures 10 **b, c**). However, the AMP_i values exhibit a slight reduction after the demixing procedure. Therefore, in cases where a quantitative evaluation of the mode amplitude distribution of localized modes is required, the demixing procedure becomes increasingly crucial.

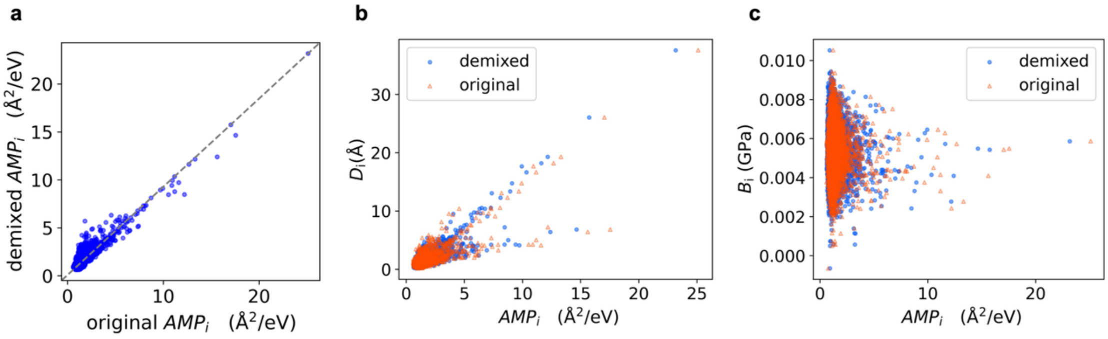

Supplementary Figure 10: Scatter plots comparing results based on original and demixed eigenvectors. **a** Comparison of AMP_i values. **b** D_i versus original and demixed AMP_i. **c** B_i versus original and demixed AMP_i.

To further investigate this correlation, we introduce a particle-based IPR based on the demixed eigenvectors as follows:

$$IPR_i = N_{atom} \frac{\sum_{p=1}^{1000} \left(\left({\psi'}_{pix} \right)^2 + \left({\psi'}_{piy} \right)^2 + \left({\psi'}_{piz} \right)^2 \right)^2}{\left(\sum_{p=1}^{1000} \left({\psi'}_{pix} \right)^2 + \left({\psi'}_{piy} \right)^2 + \left({\psi'}_{piz} \right)^2 \right)^2}.$$

This quantity characterizes the degree of vibrational localization for each particle i. ${\psi'}_{pix}$, ${\psi'}_{piy}$, and ${\psi'}_{piz}$ denote the x, y, and z components of the p-th demixed eigenvector. Supplementary Figure 11 shows the data for D_i versus IPR_i and B_i versus IPR_i. Supplementary Figure 11**a** shows a clear

correlation between D_i and IPR_i, whereas Supplementary Figure 11**b** shows no correlation between B_i and IPR_i. These results demonstrate that the localized modes are associated with the large nonaffine displacements but exhibit no correlation with the Born term.

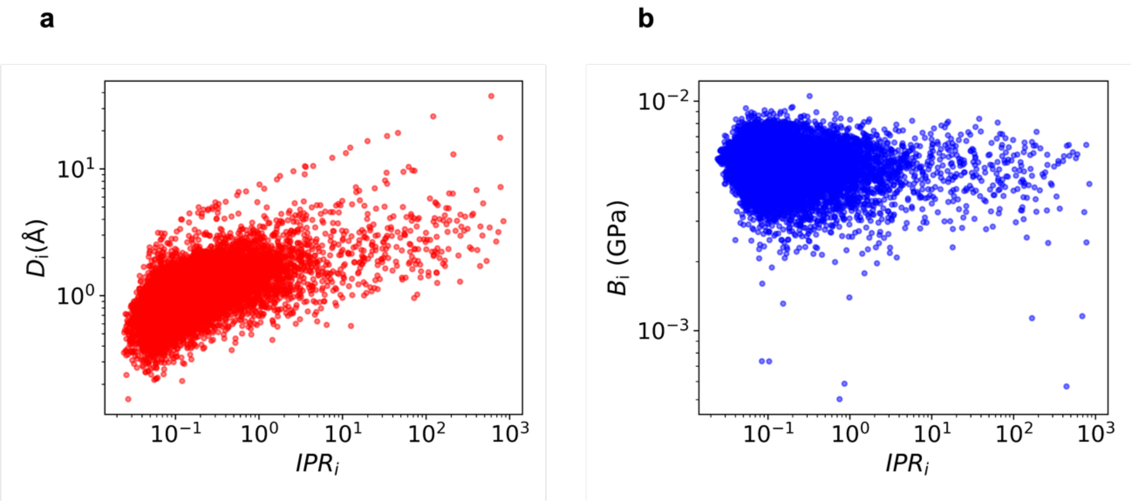

Supplementary Figure 11: Correlation between particle-based inversion participation ratio (IPR_i) and **a** nonaffine displacement D_i and **b** Born term B_i.

Dependence of structural and mechanical properties on tetrahedrality parameter in Stillinger–Weber potential

When using the Stillinger–Weber potential, the total energy is defined as

$$E = \frac{1}{2}\sum_{i}\sum_{j\neq i}\psi_2(r_{ij}) + \frac{1}{2}\sum_{i}\sum_{j\neq i}\sum_{k\neq i,j}\psi_3(r_{ij}, r_{ik}, \theta_{ijk}), \quad (1)$$

$$\psi_2(r_{ij}) = A\epsilon\left[B\left(\frac{\sigma}{r_{ij}}\right)^p - \left(\frac{\sigma}{r_{ij}}\right)^q\right]exp\left(\frac{\sigma}{r_{ij} - a\sigma}\right), \quad (2)$$

$$\psi_3(r_{ij}, r_{ik}, \theta_{ijk}) = \lambda_{SW}\epsilon\left[\cos\theta_{ijk} - \cos\theta_0\right]^2 exp\left(\frac{\gamma\sigma}{r_{ij} - a\sigma} + \frac{\gamma\sigma}{r_{ik} - a\sigma}\right). \quad (3)$$

The parameters in Equations (1)–(3) are material-dependent. For Si, the parameters are set as follows: $A = 7.049556277, B = 0.6022245584$, ϵ =2.1683 eV, $\sigma = 2.0951$ Å, $\gamma = 1.20, p = 4.0, q = 0.0$, $a = 1.80$, $\cos\theta_0 = -1/3$, and $\lambda_{SW} = 21.0$.[5] Among these parameters, λ_{SW} plays a crucial role in determining the ratio between the two-body and three-body interaction terms. A larger λ_{SW} increases the energy penalty for deviations from a perfect tetrahedral coordination in the $SiSi_4$ cluster. Therefore, λ_{SW} is referred to as the tetrahedrality parameter for atomic arrangement. Adjusting λ_{SW} allows for the simulations of other tetrahedrally bonded elements beyond silicon[6–8].

To demonstrate the generality of the results presented in this study, we conducted additional simulations using the same methodology described in the main text but with modified values of λ_{SW}, specifically λ_{SW} = 20.0 and λ_{SW} = 22.0. The cell sizes were adjusted such that the residual stress in the generated amorphous structures remained below 1 GPa, resulting in cell sizes of $L = L_x = L_y = L_z = 62.864$ Å for λ_{SW} = 20.0 and $L = L_x = L_y = L_z = 65.172$ Å for λ_{SW} = 22.0.

First, we present the structural characteristics of each set of parameters. Supplementary Figure 12 shows the calculated structure factor ($S(k)$) and radial distribution function ($J(r)$). For λ_{SW} = 21.0, the overall spectral shape in both $S(k)$ and $J(r)$ matched those in previous report[9,10]. Although better agreement with the experimental results might be achieved using the optimized Stillinger–Weber potential proposed in a recent study[11], the objective of this work was not to reproduce the detailed atomic structure of a-Si. Instead, we focused on exploring the relationship between the local structural features and mechanical responses through a persistent homology analysis. Therefore, we focused on the effects of the tetrahedrality parameter.

The principal peak at low k-values in $S(k)$ reflects the height of the $SiSi_4$ tetrahedral structure[12] shown in the inset of Supplementary Figure 12a. The first and second peaks in $J(r)$ correspond to the nearest-neighbor and second-nearest-neighbor distances, respectively. For the smaller tetrahedrality case (λ_{SW} = 20.0), the first peak of $J(r)$ is broader, and an additional peak, which is absent for λ_{SW} = 22.0 and 21.0, emerges near r = 3.2 Å. These observations indicate that the tetrahedral units are significantly distorted when λ_{SW} is reduced. Conversely, for λ_{SW} = 22.0, sharp peaks appear even in the r > 5.0 Å region, suggesting the formation of more rigid tetrahedral units, which develop MRO and microcrystalline domains.

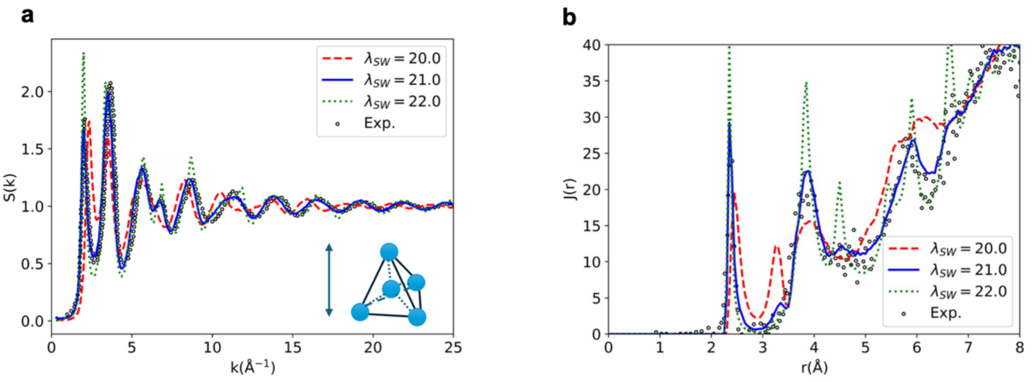

Supplementary Figure 12: Results of structural analyses for the a-Si model obtained by using λ_{SW} = 20.0, 21.0, and 22.0. **a** Structure factor $S(k)$. Open circles denote the experimental data. The inset shows a $SiSi_4$ tetrahedral unit in the a-Si structure. The position of the principal peak at low k-values is determined by the height of the tetrahedral unit. **b** Radial distribution function $J(r)$. Black open circles represent the raw data from the experimental measurements. The experimental results in **a** and **b** are taken from Refs. 9 and 10. $S(k)$ and $J(r)$ calculations were performed using the SOVA code[13].

Despite structural variations shown in Figure 9 and Supplementary Figure 12, Supplementary Figure 13 demonstrates that the lack of a clear correlation between the nonaffine deformation and Born term remains consistent across different values of λ_{SW}.

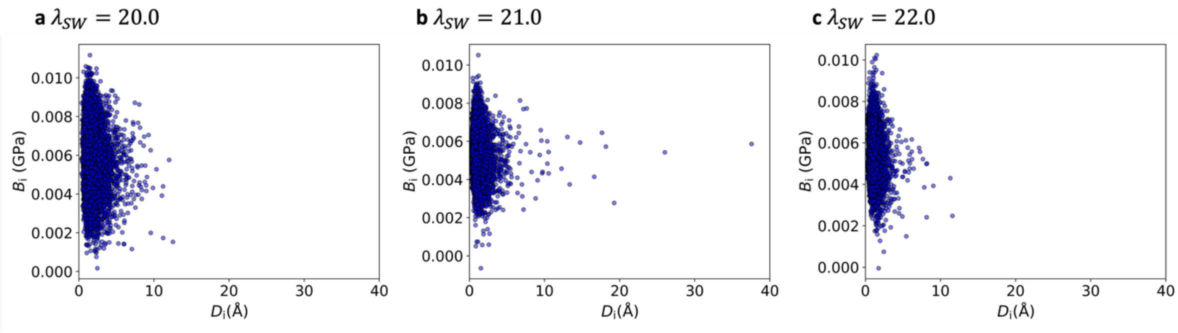

Supplementary Figure 13: Born term (B_i) and nonaffine displacement (D_i) for each atom. **a–c** Scatter plot of D_i and B_i in the structures generated with λ_{SW} = 20.0, 21.0, and 22.0.

Following the same methodology as described in the main text, we analyzed the local structural motifs associated with the top 10 atoms exhibiting the largest nonaffine displacement (NAmax10), largest Born term (Bmax10), and smallest Born term (Bmin10). Using inverse analysis of the PD, we identified rings that contained at least one atom from each of these groups and extracted the corresponding birth–death pair distributions.

Supplementary Figures 14, 15, and 16 show the birth–death pair distributions for the NAmax10, Bmax10, and Bmin10 groups, respectively. Regardless of the λ_{SW} value, the birth–death pairs in region 1 (region 2) are consistently more (less) prevalent in the NAmax10 and Bmax10 groups than in the Bmin10 group.

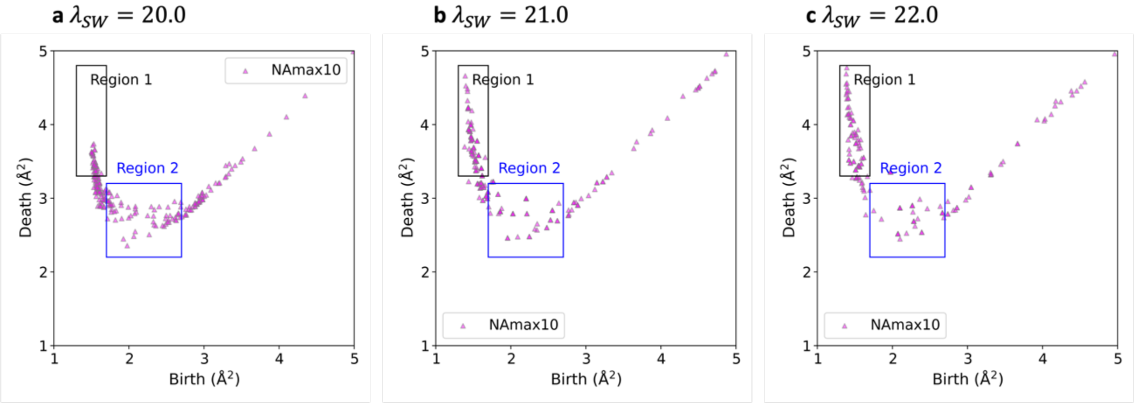

Supplementary Figure 14: Distribution of birth–death pairs for NAmax10 across different λ_{SW}. **a–c** Persistence diagrams showing the distribution of birth–death pairs for NAmax10 in the structures generated with λ_{SW} = 20.0, 21.0, and 22.0, respectively.

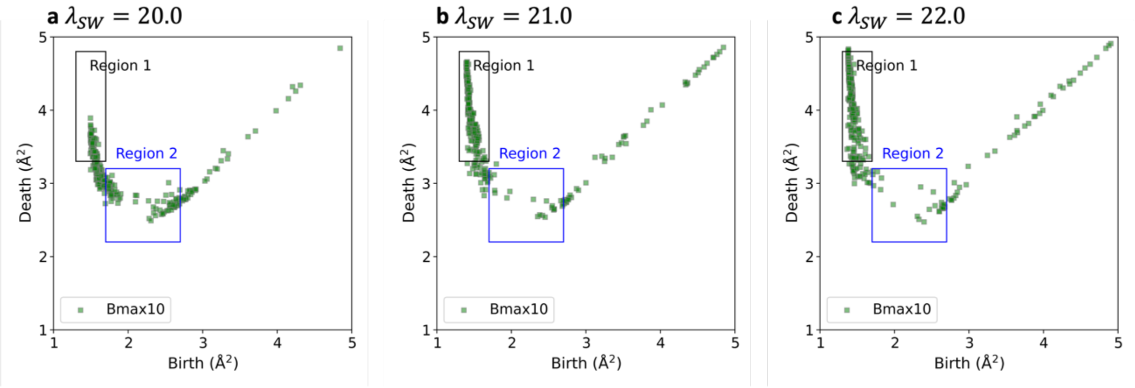

Supplementary Figure 15: Distribution of birth–death pairs for Bmax10 across different λ_{SW}. **a–c** Persistence diagrams showing the distribution of birth–death pairs for Bmax10 in the structures generated with $\lambda_{SW} = 20.0, 21.0$, and 22.0, respectively.

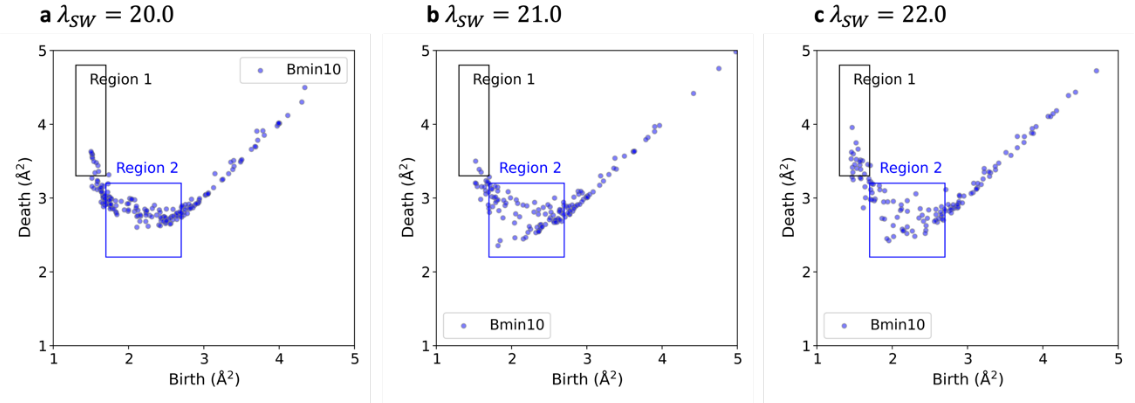

Supplementary Figure 16: Distribution of birth–death pairs for Bmin10 across different λ_{SW}. **a–c** Persistence diagrams showing the distribution of birth–death pairs for Bmin10 in the structures generated with $\lambda_{SW} = 20.0, 21.0$, and 22.0, respectively.

The percentages of birth–death pairs in regions 1 and 2 for each parameter set are summarized in Supplementary Tables 1–3.

Supplementary Table 1 Proportion of birth–death pairs in regions 1 and 2 for the structure with $\lambda_{SW} = 20.0$

**Group**	**Region 1 (%)**	**Region 2 (%)**
NAmax10	23.4	26.8
Bmax10	26.7	21.2
Bmin10	5.9	48.1

Supplementary Table 2 Proportion of birth–death pairs in regions 1 and 2 for the structure with λ_{SW} = 21.0

**Group**	**Region 1 (%)**	**Region 2 (%)**
NAmax10	44.0	20.5
Bmax10	64.1	8.1
Bmin10	2.0	53.7

Supplementary Table 3 Proportion of birth–death pairs in regions 1 and 2 for the structure with λ_{SW} = 22.0

**Group**	**Region 1 (%)**	**Region 2 (%)**
NAmax10	45.7	18.5
Bmax10	70.3	8.5
Bmin10	1.3	40.7

Furthermore, the analysis of the presence of children in the PD of the NAmax10 group indicates that irrespective of λ_{SW}, the birth–death pairs in region 1 consistently contain children. This observation supports the hypothesis that the presence of birth–death pairs in this region corresponds to the formation of the MRO (Supplementary Figure 17).

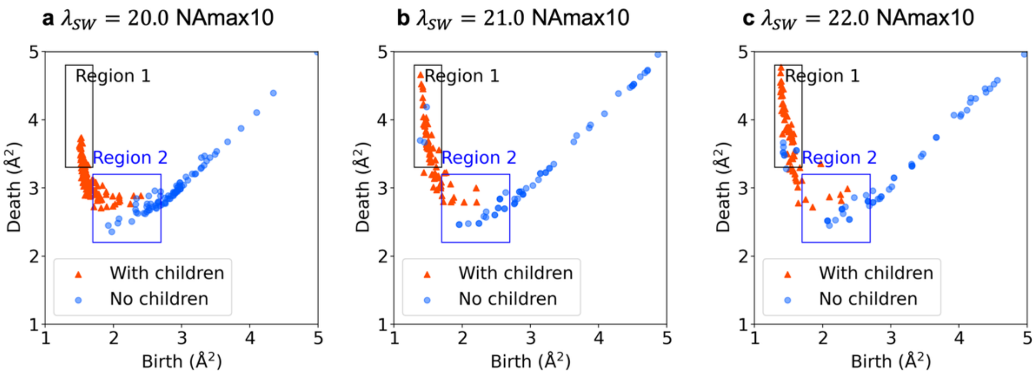

Supplementary Figure 17: Results of the child analyses. **a–c** Presence or absence of children in birth–death pairs assigned to NAmax10 in the structures obtained with λ_{SW} = 20.0, 21.0, 22.0.

We further investigated whether the surrounding structures of highly nonaffine atoms, identified via persistent homology analysis, correlated with the low-energy localized modes. Supplementary Figures 18–20 confirm that regardless of λ_{SW}, the distribution of AMP_i within the ring structures assigned for the Bmax10, Bmin10, and NAmax10 groups demonstrates that only rings in the NAmax10 group have a strong spatial overlap with regions of large amplitudes of the low-energy localized modes.

These results indicate that in covalent networks with sufficiently large tetrahedrality ($\lambda_{SW} \geq 20$), the

local structure surrounding atoms with large nonaffine displacements exhibits a hierarchical organization, where short-range disorder is embedded within the MRO. Furthermore, this structural characteristic is strongly correlated with the low-energy localized modes.

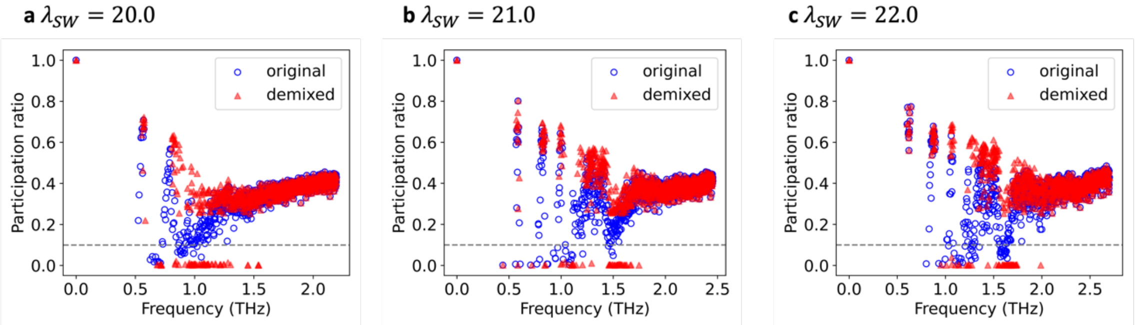

Supplementary Figure 18: Effect of demixing on participation ratio across different λ_{SW}. **a–c** Calculation results of PR as a function of mode frequency in the structures obtained with $\lambda_{SW} = 20.0, 21.0$, and 22.0. Gray dashed line indicates the threshold for identifying the low-energy localized modes. Blue open circles denote the original calculation results of PR, and red filled triangles denote the results obtained from the demixing procedure.

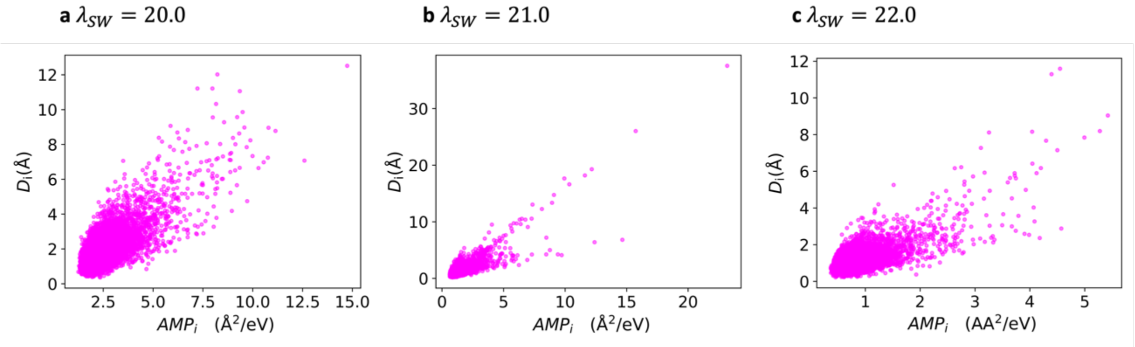

Supplementary Figure 19: Correlation between nonaffine displacement (D_i) and the amplitude of the low-energy localized modes (AMP_i) across different λ_{SW}. **a–c** D_i plotted as a function of AMP_i for the structures obtained with $\lambda_{SW} = 20.0, 21.0$, and 22.0.

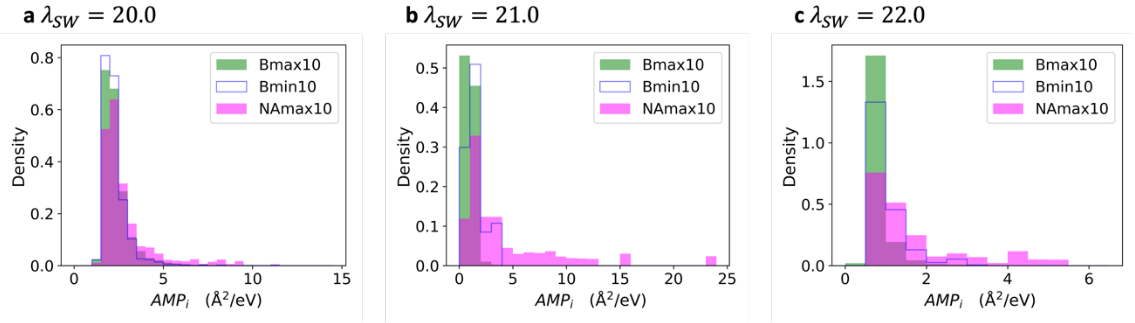

Supplementary Figure 20: Correlation between nonaffine deformation and AMP_i across different λ_{SW}.

a–c Histograms of the AMP_i values evaluated for atoms in the rings assigned to the Bmax10, Bmin10, or NAmax10 groups in the structures obtained with $\lambda_{SW} = 20.0, 21.0$, and 22.0.

Supplementary References

1. Schober, H. R. & Oligschleger, C. Low-frequency vibrations in a model glass. *Phys. Rev. B* **53**, 11469–11480 (1996).
2. Shcheblanov, N. S., Povarnitsyn, M. E., Wiles, J. D., Elliott, S. R. & Taraskin, S. N. Quasilocalized vibrations in vitreous silica. *Phys. Status Solidi B Basic Res.* **258**, 2000422 (2021).
3. Lerner, E., Düring, G. & Bouchbinder, E. Statistics and Properties of Low-Frequency Vibrational Modes in Structural Glasses. *Phys. Rev. Lett.* **117**, 035501 (2016).
4. Schober, H. R. & Ruocco, G. Size effects and quasilocalized vibrations. *Philos. Mag. (Abingdon)* **84**, 1361–1372 (2004).
5. Stillinger, F. H. & Weber, T. A. Computer simulation of local order in condensed phases of silicon. *Phys. Rev. B* **31**, 5262–5271 (1985).
6. Akahane, K., Russo, J. & Tanaka, H. A possible four-phase coexistence in a single-component system. *Nat. Commun.* **7**, 12599 (2016).
7. Dhabal, D., Chakravarty, C., Molinero, V. & Kashyap, H. K. Comparison of liquid-state anomalies in Stillinger-Weber models of water, silicon, and germanium. *J. Chem. Phys.* **145**, 214502 (2016).
8. Molinero, V., Sastry, S. & Angell, C. A. Tuning of tetrahedrality in a silicon potential yields a series of monatomic (metal-like) glass formers of very high fragility. *Phys. Rev. Lett.* **97**, 075701 (2006).
9. Laaziri, K. *et al.* High-energy x-ray diffraction study of pure amorphous silicon. *Phys. Rev. B* **60**, 13520–13533 (1999).
10. Laaziri, K. *et al.* High Resolution Radial Distribution Function of Pure Amorphous Silicon. *Phys. Rev. Lett.* **82**, 3460–3463 (1999).
11. Vink, R. L. C., Barkema, G. T., van der Weg, W. F. & Mousseau, N. Fitting the Stillinger–Weber potential to amorphous silicon. *J. Non Cryst. Solids* **282**, 248–255 (2001).
12. Salmon, P. S. & Zeidler, A. Ordering on different length scales in liquid and amorphous materials. *J. Stat. Mech.* **2019**, 114006 (2019).
13. Shiga, M. https://github.com/MotokiShiga/sova-cui.
